# Neuromodulation of Persistent Activity and Working Memory Circuitry in Primate Prefrontal Cortex by Muscarinic Receptors

**DOI:** 10.3389/fncir.2021.648624

**Published:** 2021-03-15

**Authors:** Susheel Vijayraghavan, Stefan Everling

**Affiliations:** ^1^Department of Physiology and Pharmacology, The University of Western Ontario, London, ON, Canada; ^2^Robarts Research Institute, The University of Western Ontario, London, ON, Canada

**Keywords:** muscarinic acetylcholine receptor, M1 receptor, M2 receptor, working memory, persistent activity, prefrontal cortex, antisaccade, primate

## Abstract

Neuromodulation by acetylcholine plays a vital role in shaping the physiology and functions of cerebral cortex. Cholinergic neuromodulation influences brain-state transitions, controls the gating of cortical sensory stimulus responses, and has been shown to influence the generation and maintenance of persistent activity in prefrontal cortex. Here we review our current understanding of the role of muscarinic cholinergic receptors in primate prefrontal cortex during its engagement in the performance of working memory tasks. We summarize the localization of muscarinic receptors in prefrontal cortex, review the effects of muscarinic neuromodulation on arousal, working memory and cognitive control tasks, and describe the effects of muscarinic M1 receptor stimulation and blockade on the generation and maintenance of persistent activity of prefrontal neurons encoding working memory representations. Recent studies describing the pharmacological effects of M1 receptors on prefrontal persistent activity demonstrate the heterogeneity of muscarinic actions and delineate unexpected modulatory effects discovered in primate prefrontal cortex when compared with studies in rodents. Understanding the underlying mechanisms by which muscarinic receptors regulate prefrontal cognitive control circuitry will inform the search of muscarinic-based therapeutic targets in the treatment of neuropsychiatric disorders.

## Introduction

The ability to maintain and manipulate information about the sensory world, motor actions, and previously learned experience is central to cognition and flexible behavior. Persistent, short-term elevated activity in cortical circuits has been proposed to Fuster and Alexander (1971) and Goldman-Rakic (1995) underlie the capacity to actively maintain such knowledge, or “working memory” (WM). The prefrontal cortex (PFC) in primates plays a pivotal role in the neural circuitry that processes such behaviorally relevant mental representations that are deployed to guide imminent choices and actions (Fuster and Alexander, 1971; Fuster, 1992, 1993; Miller and Cohen, 2001).

All major ascending neuromodulatory systems innervate the cerebral cortex, including the PFC and influence the dynamics of persistent activity and cortical WM circuitry (Arnsten et al., 2012). The modulatory actions of acetylcholine (ACh) on cortical function have been of long-standing interest partly because cholinergic dysfunction has been implicated in cognitive and WM deficits that manifest in psychiatric and neurological disorders including Alzheimer’s disease (Hampel et al., 2018, 2019), dementia associated with Parkinson’s disease (Noufi et al., 2019), major depressive disorder (Dagytë et al., 2011), and schizophrenia (Sarter and Bruno, 1998; Dean et al., 2003). Progressive cortical cholinergic deafferentation is a hallmark of Alzheimer’s dementia and cholinergic pathology accompanies the cognitive disruption that manifests in the disease (Hampel et al., 2018). Inhibitors of acetylcholinesterase, which breaks down released acetylcholine, is a standard component of the treatment regimen in Alzheimer’s dementia, although its efficacy in ameliorating cognitive deficits in patients has been questioned (Marucci et al., 2020). Decreased muscarinic receptor density has been reported in patients with schizophrenia (Dean et al., 2002; for detailed review see Dean et al., 2003). Further, xanomeline, a muscarinic agonist has shown clinical promise and improves short-term memory and other cognitive functions in schizophrenic patients (Shekhar et al., 2008).

Acetylcholine mediates its neuromodulatory influence via the ionotropic nicotinic and metabotropic G-protein coupled muscarinic receptor families (Picciotto et al., 2012). Subtypes from both cholinergic receptor families function in cortical WM circuitry, including in the PFC. There has recently been considerable interest in how ACh, acting through these receptors, influences neurophysiology of primate PFC during the performance of WM tasks (Baxter and Crimins, 2018; Galvin et al., 2018, 2020a; Vijayraghavan et al., 2018). Here, we will review studies of cortical muscarinic neuromodulation of WM performance and recapitulate recent work from our laboratory and others exploring muscarinic neuropharmacology of persistent activity and WM representations in primate PFC. Whereas there are several excellent published synopses regarding the functions of cortical ACh (McCormick, 1993; Steriade, 2004; Picciotto et al., 2012; Venkatesan et al., 2020), nicotinic and muscarinic neuromodulation of cognition and WM (Sarter and Bruno, 1997; Robbins and Arnsten, 2009; Klinkenberg and Blokland, 2010; Wallace and Bertrand, 2013), we will primarily focus on neurophysiological and pharmacological studies in dorsolateral PFC of non-human primates in this review.

## Localization of Muscarinic Receptors in Primate PFC

Among several cholinergic nuclei in the brainstem and basal forebrain, the nucleus basalis of Meynert is the principal source of ACh in the primate cerebral cortex (Mesulam et al., 1983; Lewis, 1991; Smiley et al., 1997). Additionally, in rodents, a fraction of cortical interneurons that express vasoactive intestinal peptide, also coexpress choline acetyltransferase, an enzyme that synthesizes ACh (Eckenstein and Baughman, 1984). However, hitherto such putatively cholinergic and GABAergic interneurons have not been shown in primate cerebral cortex (Mesulam et al., 1983), and the basal forebrain appears to be the only source of cholinergic innervation of cortex in primates. Corticopetal cholinergic afferents from the nucleus basalis innervate superficial and deep layers of macaque PFC, forming both symmetric synapses and boutons in proximity to symmetric and asymmetric synapses near dendritic spines (Mrzljak et al., 1995). Interestingly, the fraction of cortical cholinergic varicosities exhibiting synaptic specializations increases in primates when compared with rodents and is further augmented in humans (Smiley et al., 1997) versus monkeys (Mrzljak et al., 1995). Thus, ACh innervation of primate PFC has the capacity to act through both synaptic specialization and volume transmission (Mrzljak et al., 1995).

The prominent nicotinic receptor subtypes expressed in macaque cortex, including PFC, are α4β2 and α7 receptors (Wallace and Bertrand, 2013; Galvin et al., 2018). The muscarinic ACh family is comprised of G_*q*_-coupled M1, M3, and M5 receptors and G_*i/o*_-coupled M2, and M4 receptor families (Caulfield and Birdsall, 1998; Brown, 2010; Jones et al., 2012). Of these, M1 and M2 receptors are prominently expressed in PFC in primates. M1 receptors (M1Rs) and M2 receptors (M2Rs) are present in PFC in rodents (Levey et al., 1991), primates (Mrzljak et al., 1993; Medalla and Barbas, 2012) and humans (Scarr et al., 2009; Bubser et al., 2011; Dean and Scarr, 2016). M3 receptor expression has been examined in rats and is mainly expressed in the hippocampus and to a lesser extent in cerebral cortex, where it is absent in cortical layer III/IV (Levey et al., 1994; Bubser et al., 2011). However, M3 receptor expression has not been examined thus far in primate cerebral cortex owing to lack of selective immunohistochemical tools in primates. Expression patterns of other muscarinic receptor subtypes in monkey PFC are hitherto unknown.

In rodents, cortical M1 receptors are predominantly expressed postsynaptically (Levey, 1996) and are presumed to mediate the excitatory effect of muscarinic agonists on cortical activity in brain slices (McCormick, 1989, 1993; McCormick et al., 1993). Autoradiography using M1R- and M2R-preferring compounds suggests that M1R laminar expression in monkey PFC is present in all layers with strong bands of expression in layers III and V, while M2Rs are enriched in layer III and V/VI in the PFC, with the exception of Walker’s area 46, where the expression is predominantly in layer V (Lidow et al., 1989; Mrzljak et al., 1993).

M1 receptor expression in monkey cortex was examined with immunohistochemistry and mRNA expression in a series of studies by Mrzljak and colleagues (Lidow et al., 1989; Mrzljak et al., 1993, 1996, 1998). In area 46 (dorsolateral PFC) of the macaque, M1Rs were expressed throughout all layers with greatest expression found in supragranular layer III and infragranular layers V/VI. Conspicuous M1R expression was found in the soma, dendrites, dendritic spines, and in close association with both asymmetric (presumably glutamatergic) and symmetric synapses (presumably GABAergic or cholinergic). The presence of M1Rs in conjunction with asymmetric synapses in PFC circuitry points to a role in modulating thalamocortical and corticocortical excitatory transmission. Early studies indicated that M2Rs serve as autoreceptors on cholinergic efferents, inhibiting ACh release from terminals (Dudar and Szerb, 1969; Mash et al., 1985; Mash and Potter, 1986), and muscarinic inhibition of the release of tritiated ACh does not occur in M2R knock-out mice (Zhang et al., 2002). Further decline in cortical M2R expression in Alzheimer’s disease has been attributed to the degeneration of cholinergic afferents from the basal forebrain (Flynn et al., 1995; Levey, 1996). Remarkably, immunohistochemical examination of the cortical M2Rs in monkey PFC (Mrzljak et al., 1993, 1998) and primary visual cortex (Mrzljak et al., 1996; Disney et al., 2006; Disney and Aoki, 2008) revealed a more complex profile of M2R expression. PFC M2R expression was found in both pre-and postsynaptic specializations, and in both cases, the expression was associated with both symmetric and asymmetric synapses in pyramidal and non-pyramidal neurons (Mrzljak et al., 1993). In primary visual cortex, M2R expression forms interdigitated patches of dense and sparse expression that coincide, respectively, with interblobs and blobs defined by cytochrome oxidase staining (Mrzljak et al., 1996; Disney et al., 2006). M2R expression was more prominent in the parvocellular inferotemporal channel of the visual stream wherein neurons possess orientation tuning but lack color opponency. Thus, M2R expression in primary visual cortex constitutes an intriguing example of convergence between neuromodulatory specialization and functional segregation. Associational and cross-callosal projections also form interdigitated stripes in dorsolateral PFC (Goldman-Rakic and Schwartz, 1982; Schwartz and Goldman-Rakic, 1984; Pucak et al., 1996). However, it is hitherto unknown if muscarinic receptor expression demonstrates congruence with these hodological features in PFC. Mrzljak et al. (1993) did not comment on whether anisotropy was observed in the larger scale distribution of M1Rs or M2Rs in their reports on muscarinic receptors in dorsolateral PFC.

However, other elegant work from the Barbas group demonstrated hodological specificity in M2R expression in dorsolateral PFC (Medalla and Barbas, 2012). The cholinergic arousal system remains active during waking and rapid eye movement sleep (REM, “paradoxical sleep”), but not during the slow wave non-REM phase of sleep, in contrast to norepinephrine, the other major neuromodulatory system involved in arousal (Aston-Jones and Bloom, 1981; Lee and Dan, 2012). Additionally, while the cerebral cortex is in a deactivated state during non-REM sleep, positron emission tomography studies have shown that certain limbic prefrontal areas are reactivated earlier upon the transition to REM sleep, while dorsolateral PFC remains deactivated (Muzur et al., 2002). Muzur et al. (2002) proposed that this was due to selective cholinergic inhibition of dorsolateral PFC. Medalla and Barbas (2012) tested this hypothesis in the context of the anterior cingulate cortex (ACC; area 32) and areas 9 and 46 of the dorsolateral PFC. Cholinergic innervation of the ACC is dense in comparison to dorsolateral PFC, and the ACC sends a substantial glutamatergic projection to the latter (Johnston et al., 2007). The ACC is reactivated earlier during REM sleep, and the question remains as to how the dorsolateral PFC does not also get activated concomitantly, given the strong excitatory projection from ACC and restored cholinergic tone in the dorsolateral PFC during REM sleep. Medalla and Barbas (2012) hypothesized that the distribution of M2Rs on ACC projections and their postsynaptic targets could account for the lack of REM sleep activation of dorsolateral PFC. Using serial electron microscopy, fluorescent immunohistochemistry and pathway tracing, Medalla and Barbas (2012) examined M2R expression in area 9 of the PFC. They found that presynaptic M2R expression was enriched in the glutamatergic afferents from ACC to area 9 when compared with associational fibers from PFC area 46. These presynaptic M2Rs, when activated by ACh release in PFC area 9, would lead to presynaptic suppression of glutamate release (Kimura and Baughman, 1997), thus nullifying the strong excitatory drive from the ACC during REM sleep when ACh is being released in the cortical mantle. M2Rs were also found the dendritic shafts of putative inhibitory neurons targeted by the ACC projection, while M2Rs were localized primarily on dendritic spines of pyramidal neurons that were targets of the associational fibers from neighboring PFC area 46. PFC areas 9 and 46 share functional congruence in the generation and maintenance of persistent activity and WM in the cognitive circuitry of dorsolateral PFC, and therefore M2R postsynaptic expression in area 9 pyramidal neurons receiving inputs from area 46 may have facilitatory physiological effects. Similarly, cholinergic suppression in the rat piriform cortex occurs only in synapses associated with intrinsic projections, and not afferent inputs (Hasselmo and Bower, 1992). Thus, M2R expression shows remarkable specificity and correspondence with functional and hodological attributes.

## Neuropharmacology of Cortical Muscarinic Receptors in Arousal

The ascending reticular activating system, including the cholinergic and noradrenergic systems, regulate the sleep-arousal cycle and transitions between the different brain states (Moruzzi and Magoun, 1949; Lee and Dan, 2012). During slow-wave sleep (Non-REM sleep), thalamocortical circuitry is in a relatively quiescent slow oscillation with synchronized transitions between silent and active states (Steriade et al., 2001; Constantinople and Bruno, 2011). Signatures of this oscillation are manifest across different physiological scales: the activity of individual neurons and their membrane potential, the local field potential (LFP) and in the scalp electroencephalogram (EEG). Upon transitioning to REM sleep and the wake state, overall activity of individual neurons becomes desynchronized, with concomitant desynchronization in the other electrophysiological signatures of brain states, including the LFP and scalp EEG. The bistable activity seen during slow wave sleep, comprising of brief interludes of quiescence and activity have been termed down- and up-states (Contreras and Steriade, 1995). Isolated cortical slices can spontaneously replicate this mode of bistable activity, termed the slow oscillation (Sanchez-Vives and McCormick, 2000). Indeed, this low frequency (0.1–0.5 Hz) oscillation can be expressed in cultured random cortical networks and deafferented cortical slabs (Sanchez-Vives et al., 2017) and is a unifying feature of cortical activity under different anesthetic regimes (Lewis et al., 2012). It has been proposed that this is a default activity pattern and emergent property of cortical networks (Sanchez-Vives et al., 2017). It has also been speculated that the wake state may be akin to a persistent up-state (Constantinople and Bruno, 2011), where oscillatory transitions to quiescence do not occur. *In vivo*, the dynamics of ascending neuromodulatory arousal systems engender these transitions (Moruzzi and Magoun, 1949; Constantinople and Bruno, 2011; Jones, 2020).

The ascending brainstem and basal forebrain cholinergic systems causally contribute to regulating these physiological transitions accompanying brain state transitions (Metherate et al., 1992; Jones, 2008). A brief discussion of this subject may be useful here, since the physiological mechanisms that are at play in cholinergic modulation of arousal may have commonalities with cholinergic neuromodulation of cortical neurophysiology during cognitive control and WM. Interestingly, as noted previously, cholinergic nuclei are active during REM sleep, when cortical activity, LFP and the EEG are indistinguishable from the wake state, which is not the case for the monoamine arousal systems, which are very active during wakefulness, possess low activity during non-REM sleep, but are completely quiescent during REM sleep. Basal forebrain stimulation in rodents can depolarize auditory cortical neurons, desynchronize their activity with shifts in subthreshold membrane potential oscillations from low (≤5 Hz) to high (20–40 Hz) oscillations (Metherate et al., 1992). Optogenetic activation of basal forebrain cholinergic neurons, or of terminal projections thereof in the primary visual cortex, desynchronize the LFP and visual cortical neuronal activity. Lee and Dan (2012) proposed that two effects of muscarinic modulation, viz., muscarinic suppression of intracortical synaptic transmission (Gil et al., 1997; Hsieh et al., 2000; Medalla and Barbas, 2012) and muscarinic depolarization of cortical neurons may be instrumental in ACh-induced depolarization during brain state transitions. In mice, ACh neuromodulation of somatostatin-positive interneurons, that participate in a disynaptic disinhibitory relay through their inhibition of parvalbumin positive interneurons, has been shown to be necessary for the desynchronization of neuronal firing and the LFP in somatosensory cortex (Chen et al., 2015). Muscarinic agonists increase REM state duration and decrease the onset latency of REM sleep induction (Sitaram et al., 1976; Hohagen et al., 1993), while muscarinic receptor antagonists decrease the duration of REM sleep (Velazquez-Moctezuma et al., 1990; Gillin et al., 1991; Kim and Jeong, 1999). A recent study showed that a double knockout of M1Rs and M3Rs in mice almost completely abolishes REM sleep, indicating that G_*q*_ coupled muscarinic receptors were essential in regulating this epoch of the sleep rhythm (Niwa et al., 2018).

There are tonic and phasic components to the activity of cholinergic neurons and ACh fluctuations *in vivo* can be measured by amperometric methods (Parikh et al., 2004, 2007). Amperometric monitoring of ACh has revealed that tonic ACh release is coordinated across multiple brain areas during REM sleep, while phasic ACh release is synchronized during performance of a WM task (Ruivo et al., 2017).

To summarize, the cholinergic system is essential in the regulation of states of cortical arousal, and neuromodulation by muscarinic receptors, particularly of the M1R family, are essential in the generation of desynchronized states during REM sleep and awake behavior. Some of the physiological mechanisms that elicit the transitions to wake-like desynchronized cortical activity may also be involved in the neuromodulation of persistent activity in awake cortical circuits engaged in active behavior.

## Muscarinic Modulation of WM and Cognitive Control Circuitry

A substantial body of work has examined the role of the cholinergic system in cognitive performance in rodents, monkeys, and humans (Fibiger, 1991). Early clues about the importance of muscarinic receptor function in WM performance came from Bartus and Johnson (1976), who found that systemic muscarinic blockade with muscarinic antagonist scopolamine caused a delay-dependent deficit in a match-to-sample WM task, wherein the deficits were pronounced only at longer delays. Thereafter, many studies have replicated and elaborated upon this deficit in WM performance in monkeys (Rusted and Warburton, 1988; Rupniak et al., 1991; Rusted et al., 1991; Spinelli et al., 2006). Interestingly, Rupniak et al. (1991) found that the cholinesterase inhibitor, physostigmine, could reverse pro-amnestic deficits caused by systemic scopolamine, but physostigmine could not improve WM performance in aged monkeys or when distractor load was increased in the task. Moreover, they reported that stimulus luminance did not interact with scopolamine-induced deficits in delayed response performance, whereas increasing attentional load by reducing stimulus presentation time exacerbated scopolamine’s effects independent of the length of the delay, leading the authors to argue that systemic muscarinic blockade may affect attentional aspects of task performance, instead of WM. Nevertheless, a gamut of studies have shown that systemic muscarinic blockade caused WM, delayed match-to-sample, recognition memory and other cognitive deficits and pro-psychotic states (Penetar and McDonough, 1983; Aigner et al., 1987; Aigner and Mishkin, 1993; Barak and Weiner, 2006, 2009; Buccafusco et al., 2008; Plakke et al., 2008; Barak, 2009). This has led to the identification of a collection of cognitive deficits and psychosis-like symptoms termed the anti-muscarinic syndrome (Yeomans, 1995). Scopolamine has been shown to affect sensory discrimination, acoustic startle reflex, prepulse inhibition, recognition memory, short-term memory, delayed match-to-sample, set shifting, and attentional performance in rodents (Dunnett et al., 1990; Jones and Shannon, 2000; Ukai et al., 2004; Barak and Weiner, 2006). Systemic muscarinic blockade causes deficits in the WM performance and executive function in humans (Green et al., 2005; Ellis et al., 2006). Muscarinic blockade also produces deficits in the learning of rules specifying outcome-based odor discrimination in rodents (Saar et al., 2001). Lesions of the basal forebrain cholinergic system in monkeys have reported conflicting effects on cognitive tasks. In one study basal forebrain ibotenic acid lesions cause recognition memory and delayed non-match-to-sample performance deficits (Aigner et al., 1991). Contrastingly, in another study, lesions of the nucleus basalis of Meynert in monkeys appear to spare delayed response performance with short delays and instead cause attentional performance deficits (Voytko et al., 1994). Since systemic drug administration or lesions cholinergic nuclei innervating cerebral cortex could have manifold effects on the distributed brain circuitry that subserves various components of these behavioral tasks, deficits in cognitive control and WM highlighted by the studies summarized above do not necessarily indicate deficits in WM circuitry in the PFC or alterations in PFC persistent activity that maintains WM representations.

However, other reports have addressed the role of cholinergic innervation locally in the PFC in WM and other PFC-dependent cognitive tasks. Baxter’s group reported an intriguing finding: cholinergic deafferentation, by injection of an immunotoxin based on saporin in the PFC of rhesus monkeys, resulted in a specific and selective deficit in spatial delayed response, but not in other demanding tasks that engaged attention but did not require WM, such as strategy implementation, object-in-place scene learning, or reward-based decision-making as assessed by reinforcer devaluation (Croxson et al., 2011). Other evidence about the role of ACh neuromodulation during delayed response tasks comes from the neurophysiology of the nucleus basalis. In monkeys engaged in spatial delayed responses, most nucleus basalis neurons were active only during the choice and reward phases of the task, and the proportion of neurons responding during the delay period was far less prominent (Richardson and DeLong, 1986). Richardson and DeLong further reported that nucleus basalis neuronal activity in response to stimuli depended on the task context, but that the activity of these neurons was related to rewarding or aversive stimuli and cues that predict them (Richardson and DeLong, 1991). Interestingly, in macaques, intermittent stimulation of the nucleus basalis improves performance in a WM task, while continuous stimulation degrades performance (Liu et al., 2017). A brief stimulation of cholinergic fibers has been shown to cause long lasting modulation of hippocampal and cortical activity stimulation (Krnjević et al., 1981; Cole and Nicoll, 1983; McCormick and Prince, 1986), mediated by the modulation of the M-current, so named because of its inhibition by muscarinic stimulation (Brown and Adams, 1980). The M-current is generated by voltage-gated KCNQ potassium channels and it counteracts overexcitability upon neuronal depolarization; its inhibition by muscarinic receptors causes an increase in excitability of neurons.

## Muscarinic Modulation of PFC Neurophysiology and Persistent Activity

There have been fewer studies describing the neurophysiological effects of muscarinic modulation in primates. Muscarinic blockade disrupts attentional modulation of cortical activity in primary visual cortex but nicotinic blockade, while reducing V1 excitability, did not affect attentional modulation (Herrero et al., 2008). Herrero et al. (2008) also reported that muscarinic antagonist scopolamine, in addition to reducing the overall activity of PFC neurons, reduced the attentional component of V1 neuronal firing. Herrero et al. (2008) also found that ACh increased the activity of V1 neurons, and at low doses, enhanced neuronal attentional selectivity. However, at higher doses ceiling effects appeared due to non-specific increase in activity that disrupted attentional modulation. One point that emerges from this is that the actions of ACh in V1 appear to be uniformly excitatory, an observation that will be pertinent to our discussion of ACh actions in PFC later. Consistent with this effect of muscarinic receptors in attentional modulation, muscarinic receptor blockade by systemic and local infusion of scopolamine in macaque intraparietal cortex, including the lateral intraparietal area and area 7a, produced a deficit in covert orienting in a cued stimulus detection task (Davidson et al., 1999; Davidson and Marrocco, 2000). A salient stimulus appeared in one of two previously cued locations and monkeys were trained to manually respond to the stimulus onset for reward. Attention was captured by changing the luminance of one of the cues prior to the appearance of the stimulus. Analysis of reaction times after scopolamine infusion demonstrated that covert attentional orienting was compromised.

Miller and Desimone (1992) recorded neuronal activity with systemic muscarinic blockade in the inferotemporal cortex during the performance of a delayed match-to-sample recency memory task with sequential delayed presentations of multiple stimuli after a test stimulus. The rewarded response was a lever release when a succeeding stimulus matched the test stimulus. Systemic scopolamine administration was deleterious to task performance, demonstrating the ACh actions through muscarinic receptors promoted recency memory. Surprisingly, this performance deficit was not accompanied by commensurate changes in the stimulus related activity of inferotemporal neurons. The number of neurons that showed selectivity for match vs. non-match stimuli was not significantly affected by muscarinic blockade. However, when compared with placebo, scopolamine administration caused paradoxical increases in stimulus responsive neuronal activity compared with baseline activity (Miller and Desimone, 1992). This increased stimulus responsivity did not change quantifiable task-related information in the neuronal activity. Thus, it appears that the effects of muscarinic blockade on task performance were not explained by changes in inferotemporal cortical activity.

Another study examined the effects of systemic injections of scopolamine on the activity of macaque PFC neurons (Zhou et al., 2011) engaged in an oculomotor delayed response task (Hikosaka and Wurtz, 1983; Funahashi et al., 1989) with varying distractor load (Figure 2). Delay period activity was suppressed after scopolamine administration, and behavioral performance degraded (Figure 2A) with small increases in saccade reaction times and saccade end-point dispersion. Interestingly, the performance degradation due to scopolamine was independent of distractor load during the trial (Figure 2A). The effects of muscarinic blockade were contingent upon the presence of a delay, whereby visually guided saccade performance (zero second delay) was unaffected. While scopolamine had modest effects on overall neuronal activity in the PFC, it significantly affected the delay period activity after visual stimulus presentation (Figure 2B). The authors also found that stimulus-related activity during the presentation of the peripheral cue in the delayed response task was comparatively unaffected, indicating that sensory stimulus processing in the PFC was not affected. Zhou et al. (2011) also tested the effects of scopolamine on PFC activity and performance in a delayed match/non-match-to-position task. The monkeys reported whether a second cue, presented after a short delay, was at the same or different location with respect to the first cue. They found that scopolamine’s effects were not idiosyncratic to the oculomotor delayed response paradigm and manifested in the delayed match-to- position task also, whereby memory period persistent activity encoding the position of the first cue was diminished by systemic muscarinic blockade. Further, in this task, the authors found that, when the first stimulus was presented outside the neuron’s response field and the second stimulus appeared within the neuron’s response field, there was elevated activity in the delay period prior to the appearance of the second stimulus which reflected covert anticipation of the onset of the second stimulus in the response field. Muscarinic blockade diminished this anticipatory memory period activity. Thus, systemic muscarinic blockade had pronounced effects on PFC persistent activity representing the remembered location of a target and disrupted mnemonic performance.

**FIGURE 1:**
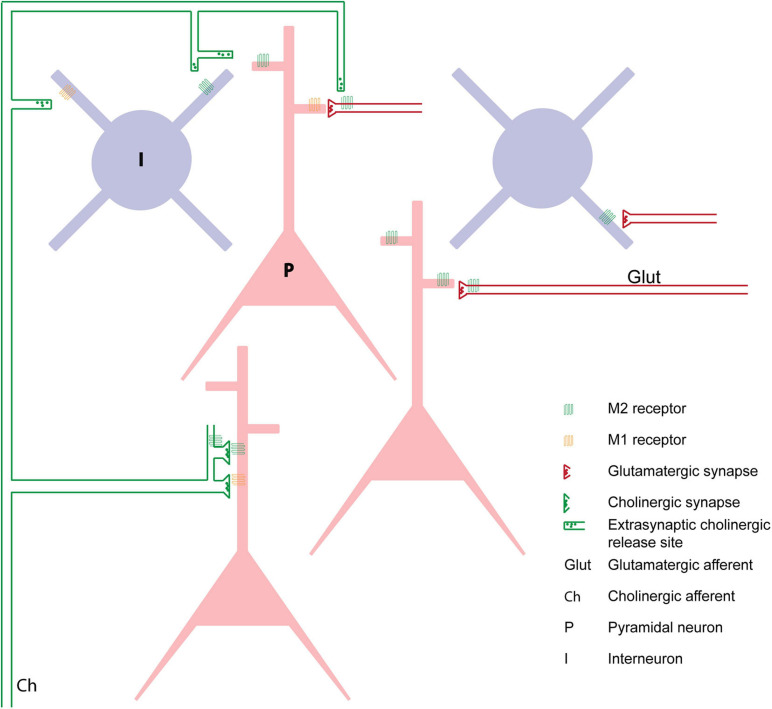
Schematic showing the localization of muscarinic receptors on PFC neurons and their relationship to cholinergic and glutamatergic innervation based on data from Mrzljak et al. (1993) and Medalla and Barbas (2012). M1Rs are localized on dendritic shafts of pyramidal and interneurons, and on the spines of pyramidal neurons where they are apposed to glutamatergic synapses. Cholinergic synapses are mainly found on dendritic shafts, while extrasynaptic cholinergic release sites on cholinergic axons result in diffuse volume transmission to influence muscarinic receptors on PFC neurons. M2Rs are found on postsynaptic dendritic spines of pyramidal neurons and on dendritic shafts of interneurons. M2Rs are also found on presynaptic terminals of glutamatergic afferents in PFC and as autoreceptors on cholinergic terminals.

**FIGURE 2:**
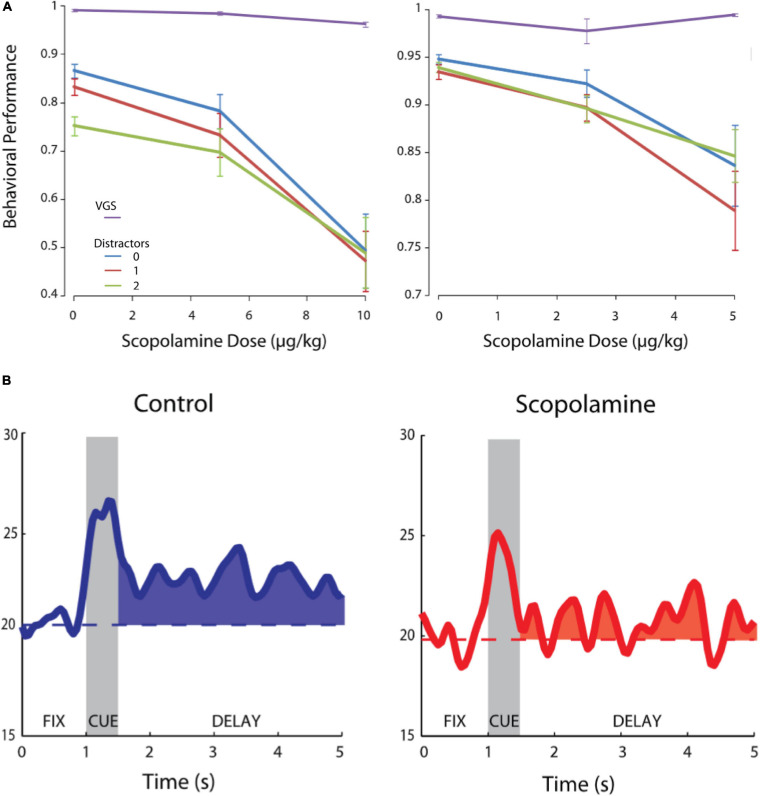
Effects of systemic scopolamine administration on oculomotor delayed response performance and delay activity of PFC units. Adapted from Zhou et al. (2011). **(A)** Behavioral performance in the oculomotor delayed response task for two monkeys after scopolamine infusion at various doses. Purple, visually guided saccades (0 s delay); Blue, 0 distractors during delay; Red, 1 distractor during delay; Green, 2 distractors during delay. **(B)** Systemic scopolamine administration reduces delay period persistent activity of PFC neurons. Modified with permission from Journal of Neurophysiology.

**FIGURE 3:**
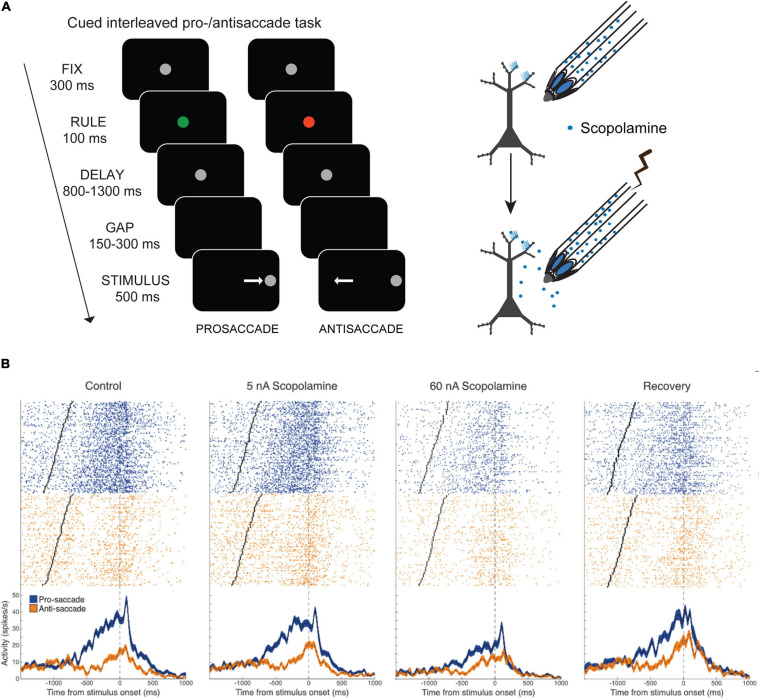
Effects of local delivery of muscarinic antagonist scopolamine by microiontophoresis on PFC activity during pro- and antisaccade task. Adapted from Major et al. (2015). **(A)** Pro- and antisaccade task structure is shown. After central fixation, the fixation spot changes to a colored rule cue which flashes briefly. Subsequently the spot becomes white again. After a delay wherein the trial rule, based on the color of the rule cue, is maintained in WM, the fixation spot disappears, and after a brief gap, the peripheral stimulus appears left or right of the fixation spot. The subject makes a saccade toward (prosaccade) or away (antisaccade) from the spot, based on the current trial rule. Trial temporal structure are also shown. Illustration on the right shows the recording and iontophoresis technique. **(B)** Microiontophoresis of increasing doses of scopolamine cause increasing suppression of the activity of a PFC neuron that has delay-period activity preferring the pro-saccade rule. Right panel shows recovery after cessation of drug application.

The results of Zhou et al. (2011) indicated that muscarinic blockade reduces activity in PFC, but since that study employed systemic injections, it could not be determined if this effect was due to blockade of local PFC muscarinic receptors or due to network consequences of muscarinic blockade elsewhere in the brain. Our group has conducted experiments on the effects of local muscarinic blockade using microiontophoresis on PFC neuronal activity while monkeys performed randomly interleaved pro- and antisaccades (Figure 3A), where the current trial rule had to be maintained in WM (Major et al., 2015). The pro- and antisaccade task is dependent of the integrity of dorsolateral PFC (Condy et al., 2007; Koval et al., 2011), and deficits in antisaccade performance are diagnostic indicator of the integrity of the PFC (Everling and Fischer, 1998). In the version of the task employed by Major et al. (2015), the rule cue was briefly presented, and had to be remembered through the memory period (Figure 3A). Persistent activity of PFC neurons encodes the task rule through this memory period (Skoblenick and Everling, 2012; Vijayraghavan et al., 2017). Microiontophoresis employs small electrical currents to eject charged moieties and drugs from the recording electrode (Hicks, 1984). The currents employed in these *in vivo* studies are on the order of ∼100 nA and are not expected to elicit extraneous electrophysiological effects on the recorded neurons. Moreover, usually, the quantities of drugs ejected are not enough to elicit behavioral effects. Major et al. (2015) found that local stimulation of muscarinic receptors dose-dependently and monotonically suppressed the activity of a majority of PFC neurons recorded during the rule WM task performance, and concomitantly degraded all forms of task-related neuronal selectivity, including WM for the rule (Figure 3B), peripheral stimulus selectivity and perisaccadic activity. Thus, some of the effects of systemic muscarinic blockade described by Zhou et al. (2011) would appear to be explained by local blockade of muscarinic receptors in PFC. In contrast to Zhou et al. (2011), we found that peripheral visual stimulus selectivity was also reduced upon local scopolamine application. These differences could be due to concentration differences due to systemic application versus local drug ejection or differences in the behavioral task structure. In the interleaved rule-based antisaccade task, activity of PFC neurons is differentially modulated by the rule prior to the onset of the peripheral stimulus. These prestimulus activity differences are, perhaps in some respects, analogous to the anticipatory activity observed in the delayed match-to-position task in Zhou et al. (2011) and may convolve with visual stimulus responsivity accordingly.

Recently, stimulation of nicotinic α4β2 receptors was also examined in monkey PFC during spatial delayed response task performance (Sun et al., 2017). The authors found α4β2 receptor stimulation also augmented PFC delay period persistent activity in the oculomotor delayed response task and improved the memory period spatial tuning of these neurons in that period. Moreover, in a variant of that task where a distractor was presented during the delay period, α4β2 receptor stimulation shielded neuronal spatial tuning during the delay period from the effects of the distractor. α4β2 receptor stimulation did not affect sensory activity related to the peripheral visual stimulus or response-related perisaccadic activity at the end of the trial. However, α4β2 agonism also enhanced the activity of neurons that had activity related to central gaze fixation. Another report from the Arnsten group showed that iontophoretic stimulation of α7 nicotinic receptors enhanced NMDA-dependent persistent activity of PFC neurons (Yang et al., 2013). An α7 receptor agonist augmented delay period activity and spatial tuning of monkey PFC neurons during spatial delayed response, an effect which could be reversed by an α7 receptor antagonist. Interestingly, α7 receptor stimulation did not have appreciable effects on peripheral stimulus-related activity and instead facilitated and synergized with the actions of NMDA NR2B receptors on PFC neurons to influence delay period persistent activity.

Thus, the findings regarding muscarinic blockade of neuronal selectivity for the peripheral visual stimulus and perisaccadic selectivity in Major et al. (2015) contrast with analysis of the effects of α4β2 nicotinic receptor stimulation on stimulus-selective and perisaccadic neurons in Sun et al. (2017) and stimulus-selective activity after α7 receptor stimulation from Yang et al. (2013). We found that muscarinic blockade reduces selectivity for all task attributes in PFC neurons, including visual stimulus and saccade direction selectivity, having a comprehensive disruptive effect on PFC neuronal task engagement. Thus, nicotinic receptor subtypes in PFC appear to be more specialized in their actions on prefrontal circuitry that generates and maintains persistent delay activity, whereas general muscarinic receptor modulation appears to affect the gamut of observable PFC task-related activity.

**FIGURE 4:**
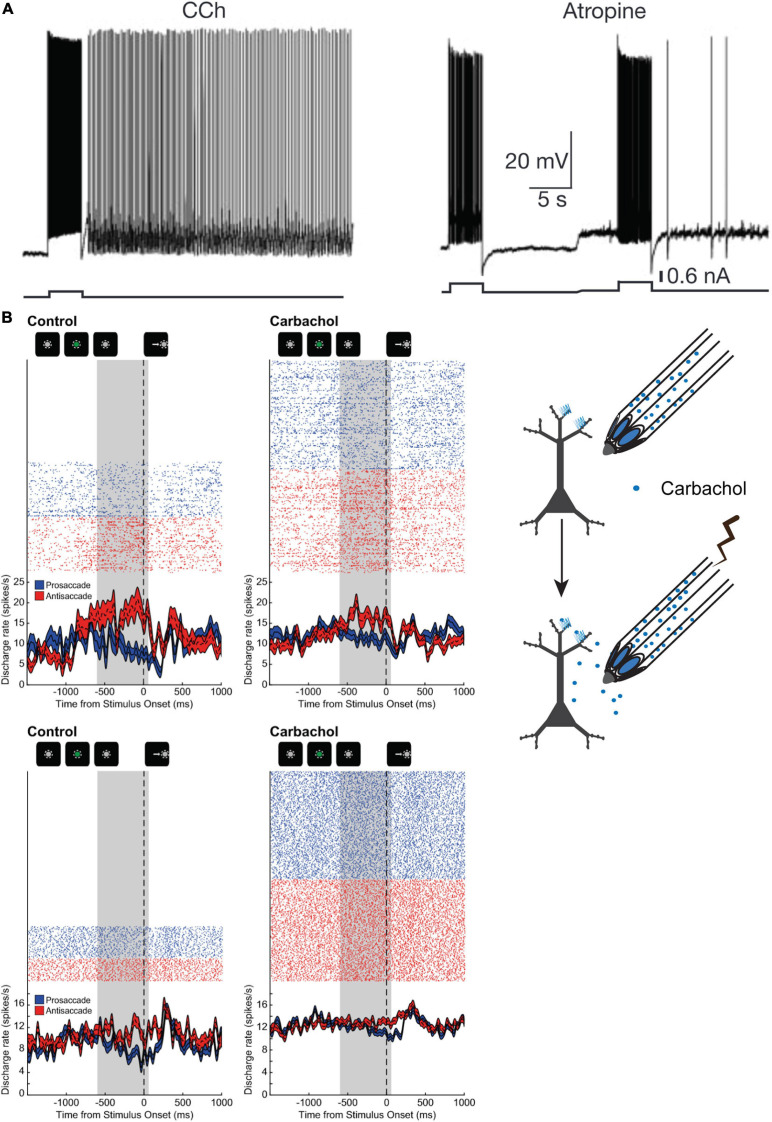
Influence of cholinergic agonist carbachol on persistent activity. **(A)** adapted from Egorov et al. (2002); **(B)** adapted from Major et al. (2018). **(A)** Example of persistent activity evoked in a rat entorhinal cortical neuron by a current pulse in the presence of carbachol. Neuronal discharge persists after cessation of stimulus, and after muscarinic blockade, and after muscarinic blockade. Neuronal discharge persists after cessation of stimulus in the presence of carbachol, but not during blockade of muscarinic receptors. **(B)** Illustration on the right shows experimental design of neuronal recording and carbachol iontophoresis. Shown on the left is the activity of two PFC neurons **(top and bottom panels)** with persistent rule-selective activity during the delay period is shown during control **(left)** and during carbachol **(right)** application. Carbachol attenuated WM activity for the antisaccade rule in the neuron shown in the **top panel**, while the activity of the neuron in the **bottom panel** was augmented by carbachol, but selectivity for the trial rule in the delay period was nevertheless diminished. Gray area shows the last 600 ms of the delay period prior to fixation offset. Reproduced with permission from Nature Publishing group.

Since muscarinic antagonism engendered such pronounced suppression of the activity of PFC neurons during WM, it would be expected that muscarinic and cholinergic agonists may enhance persistent activity and WM representations. There is some evidence that muscarinic stimulation can sustain persistent activity through intrinsic mechanisms (Egorov et al., 2002). Rat entorhinal cortical neurons, in the presence of the cholinergic agonist carbachol, respond to current pulse stimulation with long lasting activity that is reminiscent of persistent activity displayed by cortical neurons in WM tasks (Figure 4A). This carbachol-induced response is graded with increasing discharge rate after successive stimulations. The persistent responses could be blocked by general muscarinic blockade (Figure 4A) or by pirenzepine, an antagonist preferentially blocking M1Rs. Synaptic stimulation in concert with carbachol application also generated persistent spiking accompanied by the generation of nifedipine-sensitive Ca^2+^ plateau potentials. Thus, ACh, through muscarinic mechanisms, could facilitate persistent activity in cortical neurons through cell-autonomous intrinsic mechanisms that, in concert with stimulus evoked responses, could engender WM representations. However, traditionally, WM persistent activity is thought to be a network phenomenon, generated by slow reverberatory synaptic activity in a network of neurons (Wang, 2001). Whether this intriguing phenomenon that manifested in rodent entorhinal cortical slices would also occur *in vivo* in primate PFC was not clear.

To clarify whether carbachol could induce or augment persistent activity in PFC during WM task performance, our group conducted experiments where we microiontophoretically applied carbachol on PFC neurons in rhesus monkeys performing the rule-memory guided pro- and antisaccade task (Major et al., 2018). Surprisingly, we found that carbachol had mixed effects on neuronal physiology and persistent activity (Figure 4B). Carbachol application significantly excited roughly half the PFC neurons recorded, while ∼40% of neurons were inhibited. Moreover, carbachol increased the activity of broad-spiking presumed excitatory pyramidal neurons, while effects on excitability of narrow-spiking presumed mainly parvalbumin-positive interneurons were more varied. Rule encoding in the persistent activity during the delay epoch was diminished by carbachol application, especially at higher doses. This decrease in rule selectivity occurred notwithstanding the direction of changes in excitability of the neurons. It is noteworthy that carbachol is a general cholinergic agonist that has agonist activity at both muscarinic and nicotinic receptors. However, as discussed earlier, studies heretofore report that stimulation of the major nicotinic receptor subtypes in PFC appear to be generally excitatory (Yang et al., 2013; Sun et al., 2017), suggesting that the physiological actions of carbachol in Major et al. (2018) were mediated by muscarinic receptors.

## Neuromodulation of PFC Persistent Activity by Muscarinic Receptor Subtypes

Given the pervasiveness of scopolamine-induced suppression of PFC neurons described above (Major et al., 2015), and that some or all of the effects of PFC carbachol stimulation were mediated by muscarinic receptors (Major et al., 2018), the question arises as to which muscarinic receptor subtypes contributed to the various physiological effects on persistent activity and task-selectivity changes caused by these cholinergic manipulations.

As discussed previously, M1Rs are the dominant muscarinic receptor subtype expressed in PFC. Since they are localized postsynaptically at asymmetric synapses on dendritic spines of pyramidal neurons, M1R constitutes an attractive candidate for mediating the general suppression of PFC by muscarinic blockade. M1R stimulation inhibits the M-current and can thereby increase cortical neuronal excitability (McCormick and Prince, 1986; Marrion, 1997; Shirey et al., 2009; Young and Thomas, 2014). M1Rs and KCNQ channels are both expressed on dendritic spines and dendrites in layer III pyramidal neurons of PFC (Galvin et al., 2020b). An allosteric potentiator of M1R signaling increased the activity of medial PFC neurons in rodents *in vivo* and restored reversal learning in a transgenic model of Alzheimer’s disease (Shirey et al., 2009). A selective M1R antagonist and scopolamine both produce antidepressant actions in rodents due to actions in medial PFC (Navarria et al., 2015). M1R knockout mice have been found to have selective deficits in non-match-to-sample tasks while, surprisingly, showing performance enhancement in match-to-sample tasks, with a reduction in theta burst stimulation, and long-term potentiation in mice (Anagnostaras et al., 2003). An M1R positive allosteric modulator was found to enhance cognitive task performance in macaques, including self-ordered spatial search, and an object retrieval detour task (Uslaner et al., 2013). M1R also mediates long-term excitability changes in striatal neurons (Lv et al., 2017). KCNQ channels that generate the M-current are active near the action potential threshold (Brown and Adams, 1980), and inhibition of the M-current by pharmacological blockade of KCNQ channels increases PFC delay period activity during oculomotor delayed response (Wang et al., 2011). The M-current is dependent on PIP2 levels, which are regulated by phospholipase C, downstream of G_*q*_ signaling (Suh and Hille, 2002, 2005, 2007; Suh et al., 2006). Since M1R is coupled to G_*q*_ signaling and the inositol phosphate pathway (Popiolek et al., 2016; Maeda et al., 2019), it may be the main conduit for increasing neuronal excitability in primate PFC by inhibiting the M-current.

On the other hand, M1R could have inhibitory influences by direct activation of parvalbumin-positive interneurons (Yi et al., 2014). In rhesus macaque areas V1 and MT, the majority of parvalbumin-positive interneurons are found to express M1Rs (Disney and Aoki, 2008; Disney and Reynolds, 2014), although it is not clear if this is also the case in PFC. M1R activation leads to Ca^2+^ mobilization from intracellular stores through the IP3 receptor and this release of Ca^2+^ can transiently hyperpolarize neocortical neurons through the activation of calcium-activated SK potassium channels (Gulledge and Stuart, 2005). The transient suppression is usually followed by long lasting depolarization of the neuron. Metabotropic glutamate receptors also mobilize this IP3-receptor and SK channel-dependent mechanism to cause transient suppression of cortical neurons as well (Hagenston et al., 2008). One confound in the interrogation of subtype-selective muscarinic actions has been the lack of subtype selectivity of orthosteric muscarinic agonists and antagonists, the ACh binding motif is conserved among the receptor subtypes (Jones et al., 2012; Jiang et al., 2014). Among the older generation of orthosteric compounds, some, such as the agonist McN-A-343 and the antagonist pirenzepine have a pharmacological preference for M1Rs (Mitchelson, 2012) but are not highly subtype-selective (Giachetti et al., 1986; Davies et al., 2001). Recently, however, a new class of M1R agents have been synthesized that show pharmacological activity by binding at allosteric sites on the receptor and show considerable subtype selectivity and clinical promise (Bubser et al., 2011). These comprise allosteric agonists and antagonists, that act on non-ACh receptor sites and activate or inhibit the receptor directly, and positive allosteric modulators, that do not activate the receptor alone, but in concert with endogenous ACh can augment the ACh response.

**FIGURE 5:**
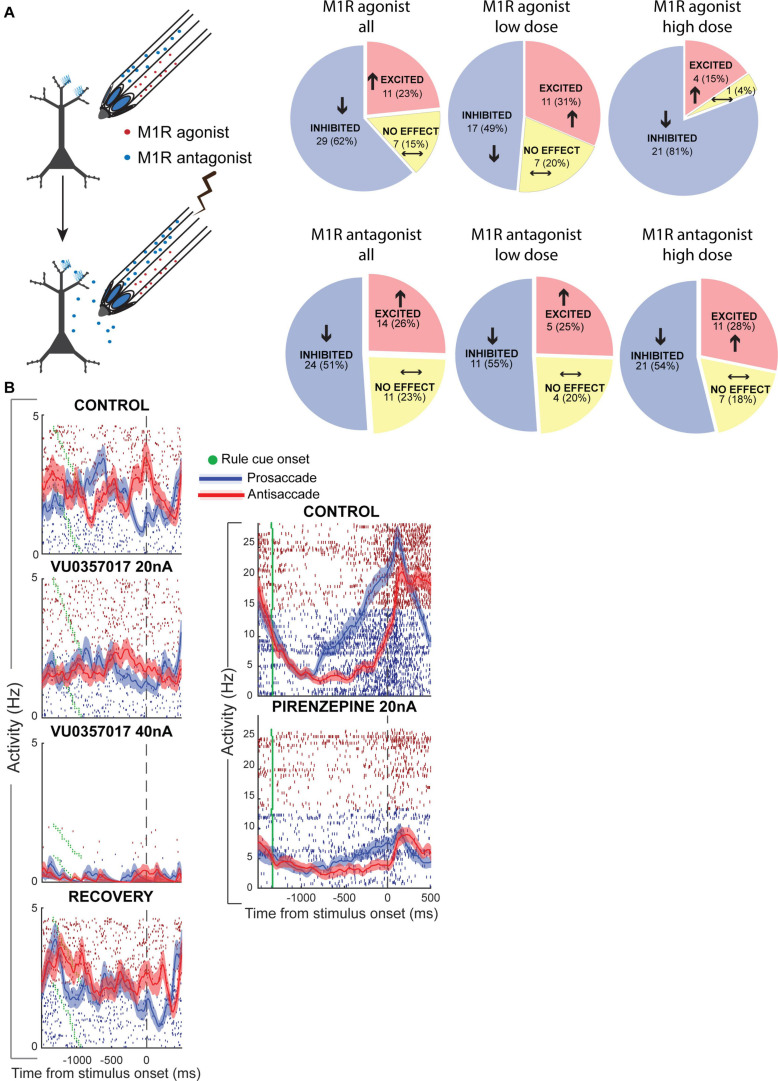
Muscarinic M1R modulation of rule WM in monkey PFC. Adapted from Vijayraghavan et al. (2018). **(A)** Experimental design of iontophoresis and recording experiments shown on the left. Effects of M1R allosteric agonist, VU0357017 **(top panel)** and M1R antagonist pirenzepine on neuronal physiology in PFC. Pie-charts show number of neurons in the population that were significantly inhibited, excited or unaffected by drug application. Left-most panel shows the net drug effect on neurons tested at any (both low and high) doses of the M1R agonist. **Middle panel**, low doses; **Right panel**, High doses. **(B)** **Left panel** shows the effects of two doses of the M1R agonist on a PFC neuron with delay period activity selective for antisaccades over prosaccades. High dose of the M1R agonist strongly suppresses the neuron and disrupts rule selectivity in the delay period. Recovery shown in **bottom left panel**. **Right panel** shows the activity of a PFC neuron before and during application of M1R antagonist pirenzepine. This neuron showed ramping persistent activity during the delay period that was selective for prosaccades. M1R blockade inhibited this neuron and also diminished rule selectivity. Modified with permission from Elsevier (Neuron).

Recently, our group has tested the effects of M1Rs on persistent WM activity for rules in monkey PFC (Vijayraghavan et al., 2018). Microiontophoresis of a selective M1R allosteric agonist, VU0357017 (Lebois et al., 2010; Digby et al., 2012), M1R-preferring agonist McN-A-343 and M1R-preferring antagonist pirenzepine were performed on PFC neurons engaged in the rule-memory guided pro- and antisaccade task described earlier (Figure 5A). Surprisingly, we found that M1R-selective allosteric agonist VU0357017 dose-dependently and strongly suppressed PFC neurons during task performance (Figure 5A). At lower dose ranges, about half of the PFC neurons tested were inhibited by the allosteric agonist, while at higher dose ranges, almost all (81%) of neurons tested were inhibited. Application of the orthosteric agonist, McN-A-343, also induced substantial suppression of ∼60% of PFC neurons. Furthermore, application of the allosteric M1R agonist disrupted the rule selectivity of the persistent delay activity of many PFC neurons (Figure 5B), while a few neurons showing increases in persistent activity and increase in rule representation (Vijayraghavan et al., 2018). Interestingly, M1R blockade with pirenzepine (Figure 5B) also suppressed the activity of many PFC neurons (Vijayraghavan et al., 2018). However, the proportion of neurons that displayed suppression did not increase with higher doses and, at the population level was pirenzepine induced suppression was milder than that observed previously with general antagonist scopolamine and milder than the suppression with the high doses of the M1R-selective allosteric agonist. Moreover, in contrast to the effects of scopolamine, although application of the M1R-selective antagonist altered the rule-selectivity in the delay period activity in some individual PFC neurons, the rule selectivity at the level of the population was not significantly altered. M1R stimulation did not differentially affect narrow-spiking putative interneurons and regular-spiking putative pyramidal neurons, indicating that increased inhibition from parvalbumin-positive interneurons could not explain the physiological suppression caused by the agonist or antagonist.

In summary, these results indicated that M1R blockade could not account for the pervasive neuronal suppression and general disruption of task selectivity that was observed with general muscarinic blockade with scopolamine (Major et al., 2015). Further, M1R overstimulation unexpectedly has strong suppressive effects on WM activity in PFC.

**FIGURE 6:**
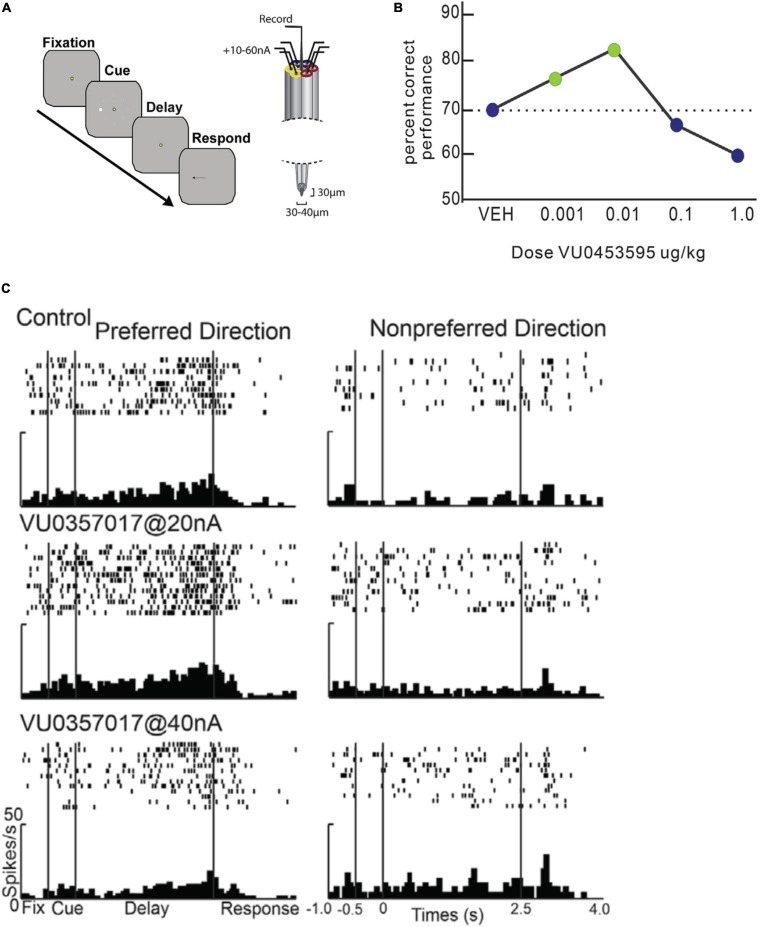
Effects of M1R stimulation on spatial delayed response performance and delay period persistent activity. Adapted from Galvin et al., 2020b. **(A)** Schematic of trial structure of oculomotor delayed response and iontophoresis technique from Galvin et al. (2020b). After central fixation, a peripheral cue briefly flashes at one of eight locations. The cue location is maintained in WM during the delay period, when central fixation continues to be maintained. At the end of the delay indicated by fixation spot offset, the subject makes a saccade to the remembered location. **(B)** Behavioral dose response curves for systemic administration of M1R positive allosteric modulator VU0453595 during spatial delayed response performance in by an aged monkey. M1R stimulation has an inverted-U effect on WM performance. WM performance degrades at doses higher than the optimal dose. **(C)** Microiontophoresis of increasing doses of M1 allosteric agonist, VU0357017 on persistent spatially tuned delay period activity of a PFC neuron. **Left panel** shows rasters and histograms for neurons preferred direction. **Right panel** shows rasters and histograms for neurons non-preferred direction. M1R agonist application at low dose enhances WM activity, while higher dose application suppresses the neuron. Reproduced with permission from Elsevier (Neuron).

Another recent study examined M1R modulation of PFC WM activity during oculomotor delayed response performance in aged monkeys (Galvin et al., 2020b). This study also found that high doses of the same allosteric M1R agonist, VU0357017, suppressed PFC WM activity, but in contrast with Vijayraghavan et al. (2018), low doses of the allosteric agonist enhanced PFC persistent activity (Figures 6A,C). Galvin et al. (2020b) also reported that systemic administration of another M1R positive allosteric modulator in aged monkeys improved WM behavioral performance at low doses but disrupted performance at high doses (Figure 6B). They further reported that inhibiting the M-current could restore delay-related firing which had been suppressed by selective M1R antagonist, telenzepine.

The effects of different muscarinic actions on neuronal physiology in the PFC from the studies discussed above have been summarized in Table 1. These surprising results with M1R agonists point to the possibility that M1R overstimulation in primate PFC may trigger signaling mechanisms that lead to neuronal suppression. Thus, the actions of ACh in PFC in alert behaving primates may involve mechanisms that engender non-trivial suppression of cortical activity through M1Rs. Further the results in Vijayraghavan et al. (2018) suggest that the actions of ACh on M1R do not completely account for the suppressive effects of general muscarinic blockade on PFC neurons.

**TABLE 1:** Qualitative comparison of the physiological effects of local muscarinic receptor manipulation on PFC WM activity from various reports discussed in this review.

Study	Muscarinic manipulation	Species	Behavioral task	Proportion of neurons significantly suppressed or excited	Notes
Major et al. (2015)	General muscarinic blockade (Scopolamine)	*Macaca mulatta*	Rule WM pro- and antisaccade task	57% **−−−** 15% **+++**	General suppression, disruption of rule, stimulus location and saccade direction selectivity
Major et al. (2018)	Cholinergic stimulation (Carbachol)	*Macaca mulatta*	Rule WM pro- and antisaccade task	39% **−−−** 49% **+++**	Rule selectivity degradation due to non-specific increase in activity
Vijayraghavan et al. (2018)	M1R stimulation (VU0357017, McN-A-343)	*Macaca mulatta*	Rule WM pro- and antisaccade task	23% **−−−** 62% **+++**	81% neurons inhibited at higher doses, with disruption of rule selectivity
	M1R blockade (Pirenzepine)			51% **−−−** 26% **+++**	Suppression does not increase with dose, and population rule selectivity not affected.
Galvin et al. (2020b)	M1R stimulation (VU0357017, cevimeline)	*Macaca mulatta* (aged)	Oculomotor delayed response task	**+++**	Low doses augment PFC WM activity in aged macaques
	M1R blockade (Pirenzepine, telenzepine)			**−−−**	Pirenzepine and Telenzepine suppress PFC WM activity

Several mechanisms may account for the suppression due to M1R overstimulation. One possibility is that M1R excitation of interneurons at high doses of stimulation leads to a suppression of PFC neurons. However, this is unlikely, as noted above because Vijayraghavan et al. (2018) reported that narrow-spiking putative parvalbumin positive interneurons were also equally suppressed by the agonist. This, of course, does not account for other classes of interneurons which are not narrow spiking, the increase in activity of which may well have caused suppression of the pyramidal neurons. Another possible mechanism for the inhibition may be SK potassium channel activation by intracellular Ca^2+^ mobilization due to M1R stimulation, as discussed elsewhere in this review (Gulledge and Stuart, 2005). It is noteworthy, that previous iontophoretic studies examining G_*q*_ protein-coupled receptors have found that stimulating these receptors has inhibitory effects on PFC neurons in primates and in some rodent studies. a1 adrenergic receptor stimulation suppresses delay period activity in a spatial delayed response task (Birnbaum et al., 2004) and G_*q*_ metabotropic glutamate receptor 1 was shown to increase inhibitory transmission in rat medial PFC, impairing decision-making (Sun and Neugebauer, 2011).

Galvin et al. (2020b) propose that suppression due to overstimulation of M1Rs could be the result of membrane hyperpolarization due to increase in the open state of KCNQ2 channels (Jentsch, 2000) due to M1R-mediated protein kinase C-cyclic AMP-protein kinase A signaling. Indeed, they show that retigabine, a positive allosteric modulator that preferentially targets KCNQ2 channels and increases the open state of the channels reduces persistent activity of PFC neurons. Future experiments must address the underlying mechanism involved in the suppression of persistent activity in the PFC by M1R overstimulation.

Vijayraghavan et al. (2018) also reported that M1R antagonist application suppressed roughly half of the PFC neurons tested even at the highest doses tested and did not systematically alter WM rule selectivity, in contrast with the uniform neuronal suppression and loss of task selectivity due to scopolamine (Major et al., 2015). This suggests that there are other muscarinic excitatory mechanisms independent of M1Rs active in the PFC. Vijayraghavan et al. (2018) proposed that there may be excitatory mechanisms based on M2R activation which may explain why M1R blockade does not replicate the efficacy of general muscarinic blockade in neuronal suppression and task selectivity. As discussed in this review, M2R is present postsynaptically in both pyramidal neuron dendritic spines and in the dendrites of interneurons. M2Rs are G_*i/o*_-coupled receptors, and previous microiontophoretic studies in monkey PFC have shown that stimulation of the dopamine D2 receptor, which is also coupled to G_*i/o*_, can augment the activity of specific classes of PFC neurons during WM tasks (Wang et al., 2004; Vijayraghavan et al., 2016, 2017; recently reviewed by Ott and Nieder, 2019). Thus, in addition to their documented role in autoinhibition and heteroinhibition as presynaptic receptors (Murakoshi, 1995), post-synaptic M2R signaling may lead to increase in PFC neuronal excitability and augmentation of persistent activity. In support of this hypothesis, preliminary data from our group suggests that M2R antagonism suppresses the delay activity of PFC cells engaged in the rule-memory guided pro- and antisaccade task. Future studies with local application of M1R-selective allosteric antagonists, like VU0255035, and M2R-selective agonists and antagonists in PFC will help resolve these apparent paradoxes of muscarinic actions on PFC WM circuits.

These results with M1R compounds in monkey PFC are of particular interest, because M1R-selective agents are being actively investigated for cognitive enhancement and amelioration of cognitive deficits in neuropsychiatric disorders (Bubser et al., 2011; Thiele, 2013; Carruthers et al., 2015). M1R based therapeutics, such as KarXT, a coformulation of M1R agonist xanomeline and trospium, a peripheral muscarinic M2R antagonist that ameliorates non-target side effects of xanomeline, are showing promising results in clinical trials for the treatment of schizophrenia (Brannan et al., 2019).

## Conclusion

In this review, we have discussed the neuromodulatory influence of the corticopetal cholinergic system through muscarinic receptors on primate PFC WM circuits that manifest persistent memory-related activity. The anatomical localization of these receptors shows exquisite specificity and correspondence with network connections within the PFC. Cortical muscarinic receptors play a pivotal role in arousal and brain state transitions, and their activation is necessary for the proper functioning of recurrent circuits in the PFC that generate persistent activity in WM tasks. Recent work shows that their role in primate PFC may be quite different from what would be expected from prior studies in other model systems like rodents and moreover, diverges from their role in sensory cortical areas. Further elucidation of muscarinic neuromodulation of PFC cognitive circuitry promises to be a rewarding endeavor for translational research and the development of new targets for the treatment of neuropsychiatric and neurological disorders.

## Author Contributions

SV and SE wrote and edited the manuscript. Both authors contributed to the article and approved the submitted version.

## Conflict of Interest

The authors declare that the research was conducted in the absence of any commercial or financial relationships that could be construed as a potential conflict of interest.
